# Bromodomain-containing factor GTE4 regulates Arabidopsis immune response

**DOI:** 10.1186/s12915-022-01454-5

**Published:** 2022-11-13

**Authors:** Qin Zhou, Yingnan Sun, Xiwang Zhao, Yue Yu, Weijia Cheng, Li Lu, Zhaohui Chu, Xiangsong Chen

**Affiliations:** 1 State Key Laboratory of Hybrid Rice, College of Life Sciences, Wuhan University, Wuhan, 430072 China; 2 Hubei Hongshan Laboratory, Wuhan, 430070 China; 3 Key Laboratory of Combinatorial Biosynthesis and Drug Discovery (Ministry of Education), School of Pharmaceutical Sciences, Wuhan University, Wuhan, 430071 China

**Keywords:** Immune response, Jasmonic acid, Bromodomain, Arabidopsis

## Abstract

**Background:**

Plants are continuously challenged with biotic stress from environmental pathogens, and precise regulation of defense responses is critical for plant survival. Defense systems require considerable amounts of energy and resources, impairing plant growth, and plant hormones controlling transcriptional regulation play essential roles in establishing the appropriate balance between defense response to pathogens and growth. Chromatin regulators modulating gene transcription are broadly involved in regulating stress-responsive genes. However, which chromatin factors are involved in coordinating hormone signaling and immune responses in plants, and their functional mechanisms, remains unclear. Here, we identified a role of bromodomain-containing protein GTE4 in negatively regulating defense responses in *Arabidopsis thaliana*.

**Results:**

GTE4 mainly functions as activator of gene expression upon infection with *Pseudomonas syringe*. Genome-wide profiling of GTE4 occupancy shows that GTE4 tends to bind to active genes, including ribosome biogenesis related genes and maintains their high expression levels during pathogen infection. However, GTE4 is also able to repress gene expression. GTE4 binds to and represses jasmonate biosynthesis gene *OPR3*. Disruption of *GTE4* results in overaccumulation of jasmonic acid (JA) and enhanced JA-responsive gene expression. Unexpectedly, over-accumulated JA content in *gte4* mutant is coupled with downregulation of JA-mediated immune defense genes and upregulation of salicylic acid (SA)-mediated immune defense genes, and enhanced resistance to *Pseudomonas*, likely through a noncanonical pathway.

**Conclusions:**

Overall, we identified a new role of the chromatin factor GTE4 as negative regulator of plant immune response through inhibition of JA biosynthesis, which in turn noncanonically activates the defense system against *Pseudomonas*. These findings provide new knowledge of chromatic regulation of plant hormone signaling during defense responses.

**Supplementary Information:**

The online version contains supplementary material available at 10.1186/s12915-022-01454-5.

## Background

Plant diseases caused by phytopathogens are the major threats for plant survival during life cycle. To counteract the pathogens, plants have evolved two layers of innate immune systems, including pattern-triggered immunity (PTI) and effector-triggered immunity (ETI) [1]. Both immune systems trigger a series of cellular response, such as burst of reactive oxygen species, programmed cell death, and upregulation of defense-responsive genes [2]. However, activations of defense responses generally consume large amount of energy and resources, thus impair plant growth. This phenomenon is termed as “growth/defense tradeoff” [3]. Recent views hypothesize that the coordinated resources allocation is the basis of growth/defense tradeoff regulation [4]. Furthermore, multi-omic studies have revealed that phytohormone crosstalk and transcriptional regulation are critical for allocating resources to coordinate growth/defense balance [5]. However, the detailed mechanisms that how phytohormones and transcriptional regulators regulate growth/defense balance are not fully understood.

Jasmonic acid (JA), one of the most studied phytohormones, plays essential roles in both plant growth and stress response [6]. Under normal condition, JASMONATE ZIM DOMAIN (JAZ) family proteins suppress the transcription of JA-responsive genes by directly inhibiting the activity of MYC transcription factors and recruiting TOPLESS repressive complex [7]. Pathogen-induced high accumulation of JA promotes the interaction between F-box protein CORONATINE INESENSTIVE 1 (COI1) and JAZs, leading to degradation of JAZs and derepression of MYCs [7]. JA signaling triggers defense response by not only activating defense-responsive gene expression but also impairing growth processes to save up energy and resource for defense. For instance, JAs generally act antagonistically against growth related hormones, including auxin, gibberellins, and brassinosteroids [3]. Stress triggered over-accumulation of JA also reduces translational efficiency of mRNAs coding proteins involved in ribosomal subunits, protein metabolism and other growth related pathways [8]. Stress-induced deficient ribosome biogenesis is also observed in other species [9], indicating that compromised ribosomal function may be a conserved approach to strengthen the stress response.

JA normally protects plants from necrotrophic fungal pathogen, such as *Alternaria brassicicola* [10]. It antagonizes to another defense-related hormone, salicylic acid (SA) which mediates the resistance to biotrophic or hemi-biotrophic pathogens, such as *Pseudomonas syringae*. pv. *tomato* (*Pst*) DC3000. However, JA and SA also act synergistically in certain circumstances. JA could positively regulate ETI through a noncanonical pathway to defend Arabidopsis plants against biotrophic pathogens [11]. JA could also enhance plant resistance to bacterial pathogen in rice [12], which may be due to the common defense system that activated by both SA and JA [13].

Histone modifications play critical roles in transcriptional regulation [14, 15]. Lysine acetylation is one of the best studied modifications on histone tails [16, 17]. Histone acetyltransferases (HATs) and histone deacetylases (HDACs) are responsible for depositing and removing acetyl group, respectively. Histone acetylation carries out functions through altering chromatin structure and/or recruiting acetylation-binding proteins, which could be regulatory factors or further recruit other regulators [18–21]. Acetylated lysine is mainly recognized and bound by bromodomain, which exists in various kinds of proteins, including HATs, chromatin remodelers, and transcription machineries [18, 20, 22, 23]. These proteins play key roles in many biological pathways, such as oncogenesis, immunity, stem cell reprogramming, and environmental response [24–27]. For instance, human bromodomain and extraterminal domain (BET) protein BRD4 (bromodomain-containing protein 4) plays key roles in regulating innate immune response, DNA break repair, and cancer [28–30]. These studies have made BRD4 become a therapeutic target [31]. In contrast, functional and mechanistic studies of bromodomain-containing proteins in plants are yet to be improved. In model plant *Arabidopsis thaliana*, the chromatin remodeler BRAHMA interacts with three bromodomain-containing proteins, BRD1, BRD2, and BRD13 [32, 33]. Although BRAHMA per se has a bromodomain, its chromatin association largely relies on BRD1, BRD2, and BRD13 [32, 33]. Another two bromodomain-containing proteins, AtMBD9 and NPX1, act as important components of SWR1 chromatin-remodeling complex, which deposits histone variant H2A.Z into chromatin [34]. Absence of AtMBD9 and NPX1 causes deficient H2A.Z occupancy and increased DNA methylation [34]. The bromodomain and ATPase domain-containing protein 1 (BRAT1) is also found to prevent DNA methylation and gene silencing [35]. There are 12 homologs of BRD4 in Arabidopsis*.* They are also named as global transcription factor group E (GTE) proteins [36]. GTE4 is involved in mitotic cell cycle maintenance during development, while GTE6 is participated in ensuring proper leaf development [36–38]. GTE1/IMB1, GTE9/BET9 and GTE11, are involved in abscisic acid signaling [26, 39]. Besides the aforementioned genetic analysis, however, deep insights of functions and mechanisms of GTE proteins, especially in regulating plant hormone signaling in immune response are absent.

In this study, we provide evidences showing that bromodomain-containing protein GTE4 negatively regulates plant immune response through repressing JA biosynthesis. Genome-widely, GTE4 binds to highly-expressed genes. The overall expression levels of GTE4-bound genes are decreased in *gte4* mutant, indicating that GTE4 mainly functions as a transcriptional activator. Within the GTE4-bound genes, we identified numerous ribosome biogenesis related genes that are downregulated in *gte4* during pathogen infection, suggesting that GTE4 tends to maintain active cellular activity under pathogen stress. GTE4 also functions as repressor of gene expression. A 12-oxophytodienoate reductase gene *OPR3*, required for JA biosynthesis, is bound and repressed by GTE4. *GTE4* disruption-resulted overexpression of *OPR3* leads to over-accumulation of JA and enhanced JA-responsive genes expression. Surprisingly, elevated JA content in *gte4* is coupled with downregulation of *PDF1.2a/b/c* and upregulation of *PR1*, which represent JA- and SA-mediated immune response, respectively. Consistently, *gte4* mutants show more resistance to bacterial pathogen *Pseudomonas syringe* pv. *tomato* (*Pst*) DC3000. In conclusion, we characterize a new chromatic regulator that attenuates plant defense response through decreasing JA content, which acts noncanonically to activate SA-mediated immune response.

## Results

### Knockout of *GTE4* enhances resistance to *Pst* DC3000 in Arabidopsis

**Fig. 1:**
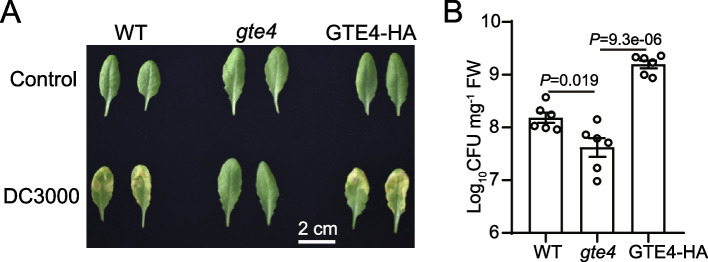
Loss-of-function *gte4* mutants are more resistant to bacterial pathogen *Pseudomonas syringae* pv. *tomato* (*Pst*) DC3000. **A** Representative leaves of WT (Col-0), *gte4* and GTE4-HA rescue plants treated with *Pst* DC3000. Photos were taken 3 days post inoculation (dpi). **B** Growth population of *Pst* DC300 in WT, *gte4* and GTE4-HA rescue plants at 3 dpi. Data are presented as mean ± SEM of 6 biological replicates. *P* values were calculated using Student’s *t*-test

In searching functions of GTE proteins in regulating plant immune system, we identified GTE4, which was shown to promote plant development in previous study [36]. Compare to wild type Col-0 (WT), *gte4* T-DNA insertion mutant (Additional file 1: Fig. S1A-C) showed weaker symptom and significant low bacteria growth population after inoculation with *Pseudomonas syringe* pv. *tomato* (*Pst*) DC3000 (Fig. 1A, B). To further confirm the phenotype, we included rescue line expressing native promoter driven *GTE4* genomic DNA tagged with C-terminal hemagglutinin tag (GTE4-HA) in *gte4* mutants (Additional file 1: Fig. S1D, E). After treated with *Pst* DC3000, we found GTE4-HA plants mostly rescued the phenotypes of *gte4* (Fig. 1A, B), suggesting that the increased disease resistance of *gte4* was indeed caused by *GTE4* disruption. Thus, GTE4 plays negative roles in resistance to *Pst* DC3000.

### GTE4 preferentially binds to highly-expressed genes involved in active cellular activity

**Fig. 2:**
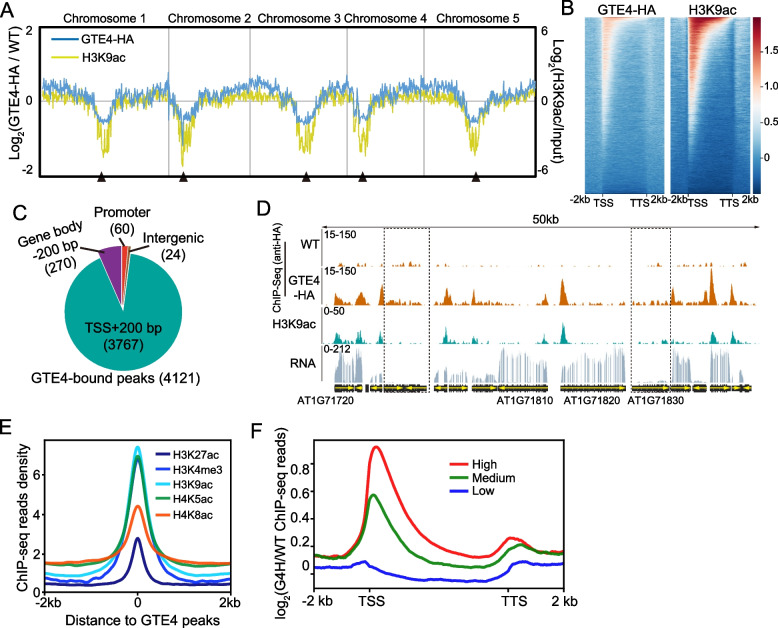
GTE4 tends to bind to active genes. **A** Chromosomal view of GTE4 occupancy. H3K9ac distribution from published data were also plotted as marker of euchromatin. Left *Y*-axis represents relative GTE4 enrichment; right *Y*-axis represents relative enrichment of H3K9ac. Black triangles indicate positions of centromere. **B** Heatmaps showing distribution patterns of GTE4 protein and H3K9ac on all genes. TSS and TTS represent transcription start site and transcription terminal site, respectively. -2 kb and 2 kb represent 2 kb upstream of TSS and 2 kb downstream of TTS, respectively. **C** Distribution of GTE4-enriched peaks. “Promoter” indicates 1 kb upstream region from TSS; “TSS + 200 bp” indicates TSS and its 200 bp downstream region; “Gene body-200 bp” indicates gene body excluding “TSS + 200 bp”; the rest of genome is defined as “Intergenic.” **D** Snapshots of IGV view of GTE4-HA ChIP-seq, H3K9ac ChIP-seq, and RNA-seq data of representative region in chromosome 1. Dashed boxes indicate lowly expressed genes with neither GTE4 binding nor H3K9ac modification. Not all loci number were listed due to space limit. **E** Metaplots showing correlation between GTE4 enrichment and typical histone modifications. ChIP-seq reads density of each modification was calculated for GTE4 peaks using published data. The summit of GTE4 peaks was set as “0,” -2 kb and 2 kb indicate 2 kb up- and down-stream of “0,” respectively. **F** Metaplots showing correlation between expression levels and GTE4 enrichment. All genes were divided evenly into 3 groups based on expression level; then, GTE4 enrichment was calculated for each group of genes. TSS, transcription start sites; TTS, transcription terminal sites; -2 kb and 2 kb represent 2 kb upstream of TSS and 2 kb downstream of TSS, respectively

To understand how GTE4 regulates immune response globally, we profiled the genome-wide occupancy of GTE4 protein by performing chromatin immunoprecipitation followed by high-throughput sequencing (ChIP-seq) using GTE4-HA and non-transgenic WT plants in parallel as a control. The overall distribution pattern of GTE4 is similar as euchromatic histone modification H3K9ac, which is enriched in euchromatic regions while depleted in centromeric heterochromatin regions (Fig. 2A, B). Total 4121 GTE4-enriched peaks corresponding to 4087 genes were identified (cutoff *P* < 1.0e − 5; Additional file 2: Table S1). Most peaks (3767 out of 4121) were located within 200 base pairs (200 bp) downstream of transcription start site (TSS) of genes (Fig. 2C, D). Given that bromodomain of GTE4 protein is a potential lysine acetylation binding module, we examined the correlation between GTE4 enrichment and histone acetylation deposition using published ChIP-seq data (Additional file 3: Table S2). By plotting histone modification ChIP-seq reads to GTE4-enriched peaks, we found that GTE4-enriched regions perfectly colocalized with typical histone acetylation marks on H3 and H4 tails, as well as an active histone methylation mark H3K4me3 (Fig. 2D, E). We further divided all genes in the genome into three groups based on their expression levels and calculated GTE4-enrichment levels for each group of genes. Consistent with the co-localization with active histone marks, GTE4 enrichment on genes were positively correlated with gene expression levels (Fig. 2F). In conclusion, GTE4 tends to associate with active genes.

**Fig. 3:**
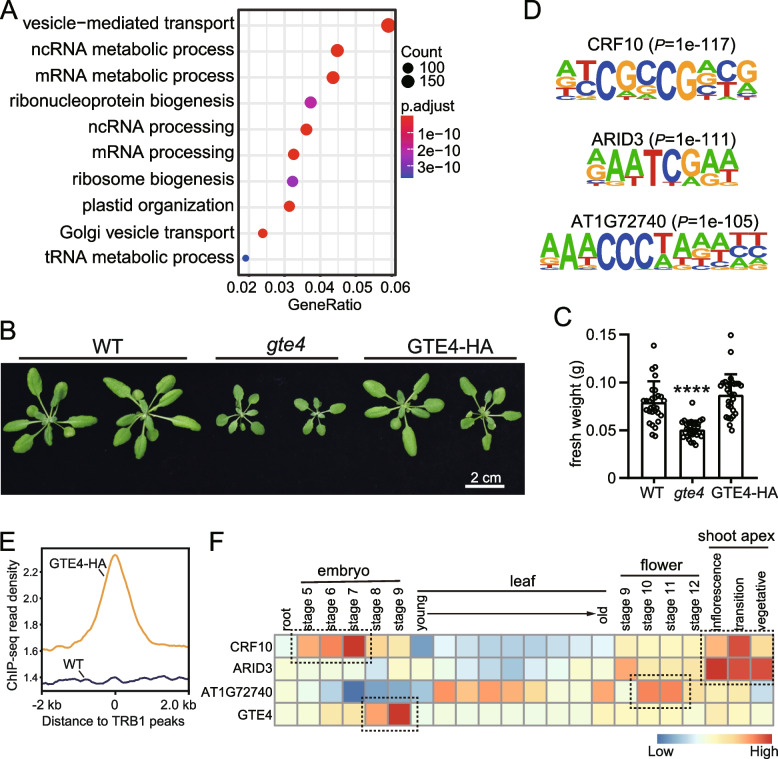
GTE4 binds to genes involved in active cellular activity. **A** GO enrichment of GTE4-bound genes. **B** Plant size of *gte4* mutant and GTE4-HA plants. Pictures were taken at 3-week-old. **C** Fresh weight quantifications of plants in (**B**). Data are presented as mean ± SEM. Student’s *t*-test, ****P < 0.001. **D** Top 3 DNA motif sequences with highest confidence identified in GTE4-enriched peaks. **E** Metaplots shows GTE4 enrichment on published TRB1 ChIP-seq peaks. **F** Heatmaps of expression patterns of *CRF10*, *ARID3*, *AT1G72740*, and *GTE4* in various tissues using published data. Dashed black boxes highlighted tissues with high expression levels

To reveal the potential functions of GTE4 targets, we firstly performed Gene Ontology (GO) analysis, and found that GTE4-bound genes were significantly enriched in vesicle-mediated transport, ncRNA (mainly rRNA) and mRNA metabolic process, and ribosomal protein biogenesis (Fig. 3A, Additional file 4: Fig. S2). Given that active cell division and tissue growth require efficient ribosome biogenesis, these data suggest that GTE4 may be involved in plant development. Indeed, previous study showed that loss-of-function *gte4* mutant displayed deficient embryo and root development [36]. We also observed smaller biomass of *gte4* mutant (Fig. 3B, C). We then searched conserved DNA motifs in GTE4-enriched peaks. The top 3 motifs with highest confidence were known as binding motifs of cytokinin response factor 10 (CRF10), AT-rich interaction domain 3 (ARID3), and a single MYB histone (SMH) family member (AT1G72740), respectively (Fig. 3D), suggesting that GTE4 may at least partially share targets with CRF10, ARID3, and AT1G72740 protein (single MYB histone, SMH). Interestingly, CRF proteins, similar as GTE4, are involved in regulation of development and stress response [40]. ARID proteins are widely involved in transcriptional regulation. SMH proteins are well known as telomere binding proteins (TRBs), which ensure proper maintenance of life span [41, 42]. Consistently, previous ChIP-seq analysis of one TRB member, TRB1, identified similar DNA motif as GTE4 [43]. We called TRB1-enriched peaks using published data [43] and calculated GTE4 ChIP-seq read density on TRB1-peaks, and found significant enrichment of GTE4 compare to WT (Fig. 3E). Interestingly, TRB1 also promotes plant growth, and binds to genes involved in ribosome biogenesis [43, 44]. Furthermore, published expression data [45] showed that *CRF10*, *ARID3*, *AT1G72740*, and *GTE4* are highly expressed in tissues with active cell division, such as flower, shoot apex, and developing embryo (Fig. 3F). Taken together, GTE4 tends to bind genes that may be involved in active plant growth.

### GTE4 promotes gene expression generally

**Fig. 4:**
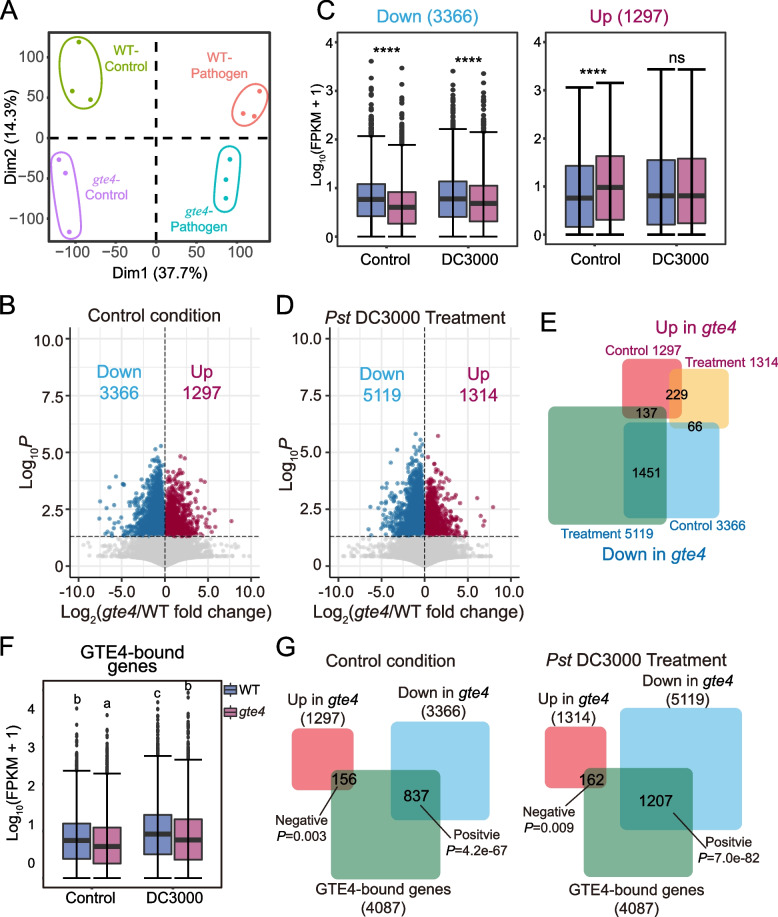
GTE4 promotes gene expression generally. **A** Principal component analysis (PCA) of RNA-seq data in WT and *gte4* under control and *Pst* DC3000 treated conditions. **B** Volcano plots showing differentially expressed genes (DEGs) in *gte4* under control condition. **C** Boxplots showing the expression levels of DEGs from **B** under *Pst* DC3000 treatment condition. FPKM, Fragments per kilo base per million mapped reads. ****P < 2.2e − 16; ns, no significance. **D** Volcano plots showing DEGs in *gte4* under *Pst* DC3000 treatment condition. **E** Venn diagrams of DEGs in *gte4* under control and *Pst* DC3000 treated conditions. **F** Box plots showing expression level of GTE4-bound genes in WT and *gte4* under control and *Pst* DC3000 treated conditions. Different lowercase letters represent significant differences by one-way ANOVA with Tukey's post hoc test among assessed samples (*P* < 0.01). **G** Venn diagram of overlapped genes between GTE4-bound genes and DEGs in *gte4* under control and *Pst* DC3000 treated conditions

In order to investigate the functions of GTE4 on gene expression during pathogen infection, we performed mRNA transcriptome analysis (RNA-seq) with WT and *gte4* mutant plants under control and *Pst* DC3000 challenged conditions with 3 biological replicates (Fig. 4A). Under control condition, we identified 1297 upregulated and 3366 downregulated genes in *gte4* (Fig. 4B, Additional file 5: Table S3). We then examined the behavior of these *gte4*-resulted differentially expressed genes under pathogen inoculation and found that the downregulated genes identified under control condition were still significantly downregulated in *gte4* upon pathogen inoculation, while the upregulated genes under control condition did not show significant difference any longer upon pathogen challenge (Fig. 4C), indicating that the downregulated genes are more likely determined by *gte4* genotype, while upregulated genes largely depend on stress conditions. Specifically, we identified 1314 upregulated and 5119 downregulated genes upon pathogen inoculation in *gte4*, respectively (Fig. 4D, Additional file 6: Table S4). Consistent with previous observations, large proportion of downregulated genes under control condition (1451 out of 3366, 43.1%; Fisher’s exact test, *P* = 2.7e − 285) were also found downregulated under pathogen inoculation (Fig. 4E). In contrast, the upregulated genes under two conditions showed an overlap with much less proportion (229 out of 1297, 17.7%) (Fig. 4E). These results suggest that the downregulation of genes may be directly caused by *GTE4* disruption. To confirm that, we integrated the GTE4 ChIP-seq data with RNA-seq data and found that GTE4-bound genes were significantly downregulated in *gte4* under both control and pathogen inoculation conditions (Fig. 4F). Consistently, GTE4-bound genes showed positive overlap with downregulated genes in *gte4*, but negative overlap with upregulated genes in *gte4* under both conditions (Fig. 4G). Taken together, GTE4 tends to bind to the actively transcribed genes and maintain their transcription levels.

### GTE4 maintains active expression of ribosome biogenesis-related genes during pathogen infection

**Fig. 5:**
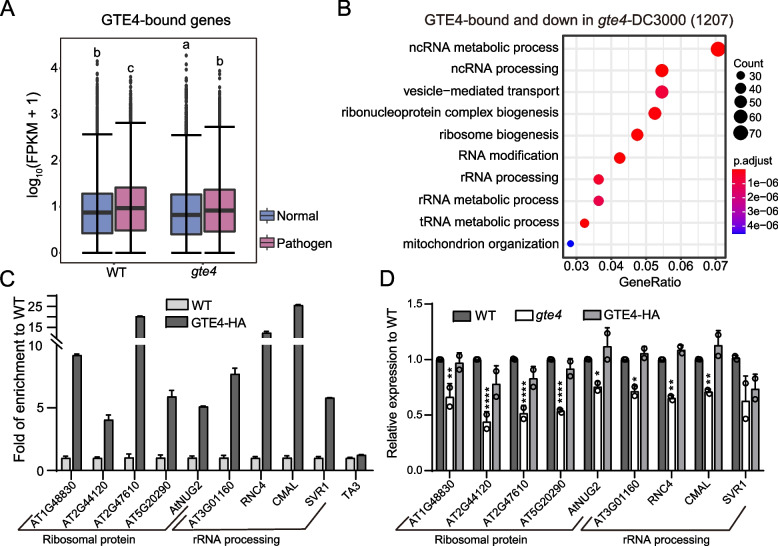
GTE4 promotes the expression of ribosome biogenesis-related genes during pathogen infection. **A** Boxplots showing expression levels of GTE4-bound genes in WT and *gte4* under control and pathogen infection conditions. Different lowercase letters represent significant differences by one-way ANOVA with Tukey’s post hoc test among assessed samples (*P* < 0.01). **B** GO analysis of genes with GTE4 binding and downregulated in *gte4* after pathogen treatment. **C** ChIP-quantitative PCR (qPCR) verification of GTE4 binding on ribosomal protein and rRNA processing related genes. Data are presented as mean ± SEM of 3 technical replicates. Transposable element *TA3* serves as negative control. **D** Reverse transcription (RT)-qPCR verification of downregulation of ribosomal protein and rRNA processing related genes. Data are presented as mean ± SEM of 2 biological replicates. Student’s *t*-test, *P < 0.05, **P < 0.01, ****P < 0.001

To reveal the relationship between GTE4-bound targets and pathogen resistance, we firstly checked the behavior of GTE4-bound genes during pathogen infection and found that the most GTE4-bound genes were significantly elevated by pathogen infection in WT, as well as in *gte4* but with a lower degree than in WT (Fig. 5A). The lower induced degree of GTE4-bound genes in *gte4* may be due to the lower basal expression levels in *gte4* before pathogen treatment (Fig. 4F, Fig. 5A). Since the major function of GTE4 is promoting gene expression, we focused on the 1207 genes that are bound by GTE4 and downregulated in *gte4* upon pathogen treatment (Fig. 4G). GO analysis showed that these genes were enriched in ncRNA (mainly rRNA) processing and ribosome biogenesis related pathways (Fig. 5B), which are required for protein translation and active cellular activities. We randomly selected 5 ribosomal protein genes and 4 rRNA processing related genes for further verification. ChIP followed by quantitative PCR (ChIP-qPCR) showed that all tested genes were bound by GTE4 (Fig. 5C). As expected, the expression levels of these genes were decreased in *gte4* mutant and mostly restored in GTE4-HA rescue plants as showed by reverse transcription (RT)-qPCR (Fig. 5D). In addition, we also validated the expression of several development-related genes chosen form the 837 GTE4-bound and GTE4-activated genes under control condition by qRT-PCR in *gte4* (Additional file 7: Fig. S3). Considering that *gte4* mutant is deficient in development (Fig. 3B), we speculate that lower expression of ribosome biogenesis-related genes in *gte4* during pathogen infection may save up more energy for plant immune system.

### Over-accumulation of JA is coupled with elevated *PR1* expression in *gte4* mutant

**Fig. 6:**
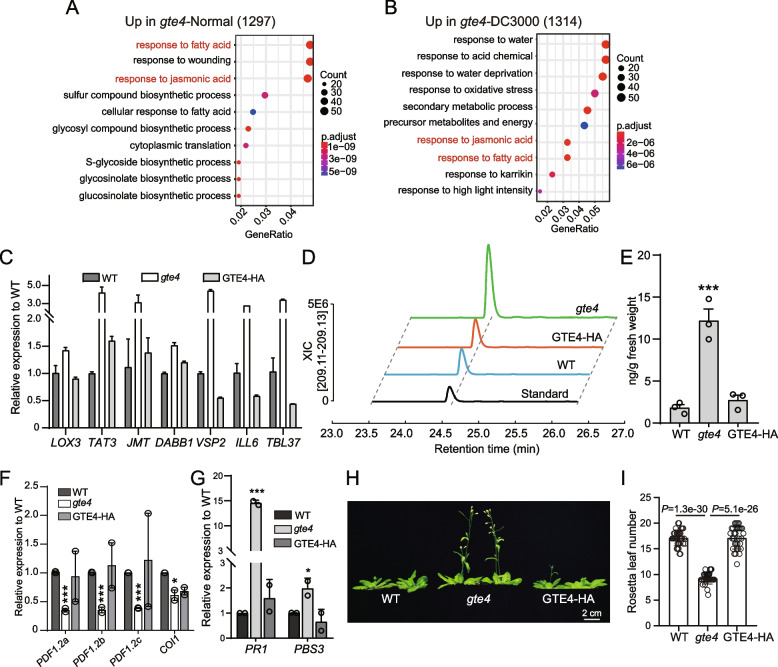
GTE4 regulates JA content and JA-, SA-responsive gene expression. **A**, **B** GO analysis of upregulated genes in *gte4* under control (**A**) and *Pst* DC3000 treatment conditions (**B**). **C** RT-qPCR validation of JA-responsive genes expression in *gte4*. Data are presented as mean ± SEM of 3 technical replicates. Another biological repeat is presented in Additional file 8: Fig. S4A. **D** Chromatograms of LC–MS data for measurement of JA content in WT, *gte4* mutants, and GTE4-HA rescue plants. XIC, extracted ion chromatograms. **E** Quantification of JA content in (**D**). Data are presented as mean ± SEM of 3 biological replicates. Student’s *t*-test, ***P < 0.005. **F** RT-qPCR of *PDF1.2a/b/c* and *COI1* expression in *gte4* and GTE4-HA plants. Data are presented as mean ± SEM of 2 biological replicates. Student’s *t*-test, ***P < 0.005. **G** RT-qPCR of *PR1* and *PBS3* expression in *gte4* and GTE4-HA plants. Data are presented as mean ± SEM of 3 biological replicates. Student’s *t*-test, ***P < 0.005. **H** Flowering phenotype of *gte4* plants. Pictures were taken at 4-week-old. **I** Rosetta leaf number of plants showed in (**H**). Data are presented as mean ± SEM of at least 30 plants

We next turned our attention to the upregulated genes in *gte4*. Total 1297 and 1314 upregulated gene were identified in *gte4* under control and pathogen treatment condition, respectively (Fig. 4B, D). Interestingly, GO analysis showed that both groups of upregulated genes were enriched in fatty acid and jasmonic acid (JA)-responsive pathways (Fig. 6A, B). We further selected 7 known JA-responsive genes that were upregulated in *gte4* under both conditions for validation by RT-qPCR. Consistent with RNA-seq data, all the tested JA-responsive genes were upregulated in *gte4* and mostly restored to wild-type level in GTE4-HA rescue lines (Fig. 6C, Additional file 8: Fig. S4A). The upregulation of JA-responsive genes in *gte4* under both conditions indicate that JA signaling, which is normally triggered by many kinds of biotic and abiotic stresses, is already activated in *gte4* mutants. In addition, given the critical roles of JA in cell cycle and stress response [46–48], higher activation of JA signaling may explain the phenotype of *gte4* mutants, including mitotic cell cycle defects [36]. Consistently, genes involved in plant growth related pathways, including chromatin organization and ribosome biogenesis, were downregulated in *gte4* under control or pathogen challenge condition, respectively (Additional file 8: Fig. S4B, C). To find out why JA-responsive gene are over-activated, we measured the JA content in *gte4* by liquid chromatography coupled with mass spectrometry (LC–MS) and found that JA level was significantly increased in *gte4* and restored in GTE4-HA (Fig. 6D, E). These results are unexpected, because JA normally functions antagonistically to SA, which induces expression of PR genes to increase plant resistance to *Pst* DC3000. Nevertheless, a few studies also reported that JA may promote SA-mediated immune pathway in a noncanonical manner [11]. To confirm that, we examined the expression of marker genes for JA-mediated immune response (*PDF1.2a/b/c* and *COI1*) and SA-mediated immune response (*PR1* and *PBS3*). We found that *PDF1.2a/b/c* and *COI1* were significantly downregulated, while *PR1* and *PBS3* were dramatically increased in *gte4* (Fig. 6F, G), suggesting that enhanced JA content in *gte4* acts through a noncanonical pathway to activate SA-mediated immune response and enhance plant resistance to *Pst* DC3000. Consistent with the noncanonical behavior of over-accumulated JA, *gte4* mutant showed early flowering phenotype than wild-type (Fig. 6H, I), while increased JA content is normally supposed to cause late flowering in Arabidopsis [49, 50]. Taken together, GTE4-regulated JA content may be involved in a noncanonical pathway to activate SA-mediated immune system.

### GTE4 inhibits JA biosynthesis by binding and repressing *OPR3*

**Fig. 7:**
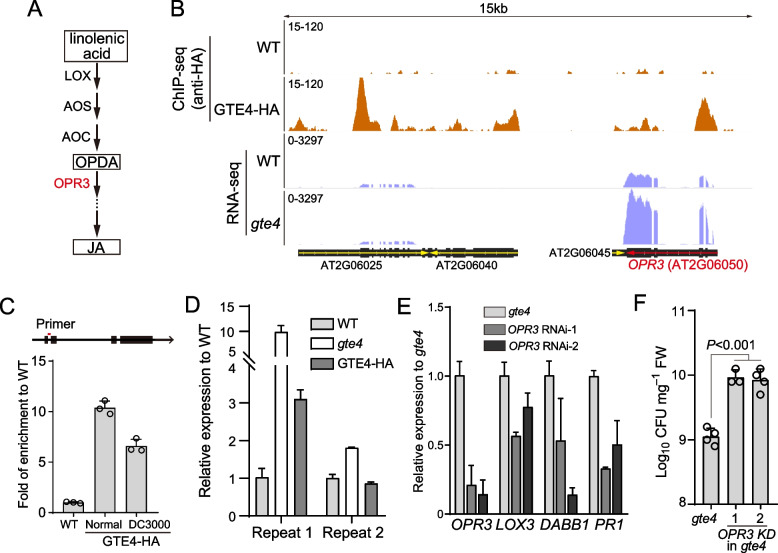
GTE4 suppresses JA biosynthesis through binding and repressing *OPR3.* **A** Brief diagram of JA biosynthesis. LOX, lipoxygenase; AOS, hydroperoxide dehydratase; AOC, allene oxide cyclase; OPDA, 12-oxophyto-10,15-dienoic acid. **B** Snapshots of IGV view of GTE4-HA ChIP-seq and *gte4* RNA-seq data of *OPR3* locus. **C** ChIP-qPCR verification of GTE4-enrichment on *OPR3*. Data are presented as mean ± SEM of 3 technical replicates. Position of primer is indicated with red lines. **D** RT-qPCR verification of *OPR3* upregulation in *gte4*. Two biological repeats are presented. Data are presented as mean ± SEM of 3 technical replicates for each biological repeat. **E** RT-qPCR showing expression of *OPR3*, JA-responsive genes *LOX3* and *DABB1*, and *PR1* expression in OPR3 knockdown in gte4 background plants. RNAi-1 and RNAi-2 indicate two independent lines. Data are presented as mean ± SEM of 2 biological replicates. **F** Growth population of *Pst* DC300 in *gte4* and 2 independent lines of *OPR3* knockdown (KD) in *gte4* background plants at 3 dpi. Data are presented as mean ± SEM of at least 3 biological replicates. *P* value was calculated using Student’s *t*-test

To further reveal how GTE4 regulates JA biosynthesis, we overlapped GTE4-bound genes with co-upregulated genes in *gte4* under both control and pathogen treatment conditions and obtained 20 genes (Additional file 9: Table S5). Among them, we identified *oxophytodienoate-reductase 3* (*OPR3*, AT2G06050), which encodes a 12-oxophytodienoate reductase required for JA biosynthesis (Fig. 7A, B) [51]. We validated the GTE4-binding on the downstream of TSS of *OPR3* by ChIP-qPCR (Fig. 7C), as well as the upregulation of *OPR3* in *gte4* by RT-PCR (Fig. 7D). To further confirm that pathogen resistance of *gte4* is due to upregulation of *OPR3*, we knocked down *OPR3* in *gte4* using artificial microRNA [52]. As expected, expression of JA-responsive genes *LOX3* and *DABB1* were decreased coupled with knockdown of *OPR3* (Fig. 7E). Furthermore, downregulation of *OPR3* in *gte4* indeed reduced pathogen resistance compare to normal *gte4* mutants (Fig. 7F). Consistently, *PR1* expression was also downregulated (Fig. 7E). Taken together, these data suggest that GTE4 counteracts pathogen resistance by attenuating JA biosynthesis through inhibiting the expression of the *OPR3*.

## Discussion

### Roles of GTE4 in coordinating plant growth and defense

**Fig. 8:**
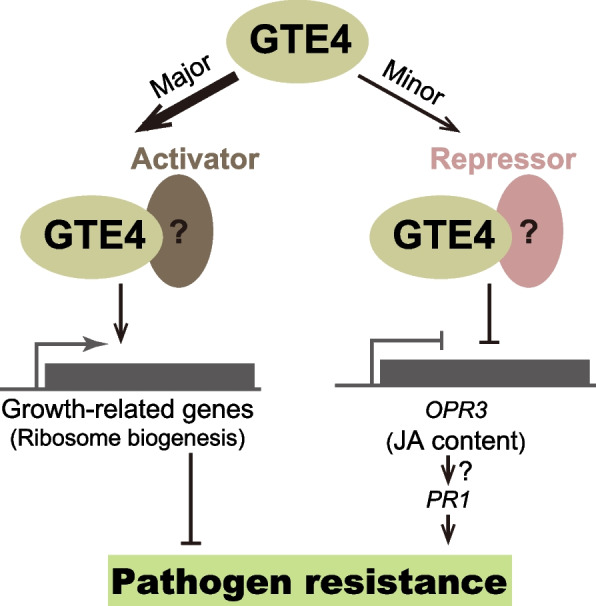
Working model of GTE4 function in regulating pathogen defense. In most circumstances, GTE4 facilitates gene expression, likely through interacting with transcriptional activators. Meanwhile, a small proportion of GTE4 may also interact with transcriptional repressors to suppress gene expression. In the case of pathogen infection, GTE4 maintains high expression of ribosome biogenesis related genes, which play negative roles in defense response due to consumption of energy and resources. Meanwhile, GTE4 reduces JA content by binding and repressing JA biosynthesis gene *OPR3*. In the absence of GTE4, increased JA content activates *PR1* expression through an unknown noncanonical pathway. Together, GTE4 negatively regulates immune response by both promoting plant growth and attenuating JA biosynthesis

Plants are subjected to constant environmental stimuli. To successfully survive from stresses, plants have evolved immune systems, which however usually cost the expense of growth. Hence, precisely regulation of growth/defense balance is critical for plants’ survival under environmental stresses. In this study, we have identified a new regulator GTE4 which pushes the balance towards growth during pathogen infection. On the one hand, GTE4 binds to ribosome biogenesis genes and maintains their high expression during pathogen infection. Given that efficient ribosome biogenesis is usually required for active cellular activity and consumes most of the energy in cells [53], GTE4 may motivate the plant to consume too much energy and resources for growth instead of defense response. This could also explain the deficient cell cycle procedure and development phenotypes of *gte4* mutant [36, 37]. On the other hand, GTE4 represses *OPR3* to attenuate JA biosynthesis and restrict stimulated defense response. Disruption of *GTE4* results in overaccumulation of JA and enhanced JA-responsive gene expression, and subsequently enhanced resistance. Furthermore, JA is believed to play fundamental roles in regulating growth/defense balance, by not only activating defense system, but also impairing auxin- and brassinosteroids-mediated growth pathways [3]. Together, GTE4 plays negative roles in immune response through both promoting plant growth and attenuating JA biosynthesis (Fig. 8).

Consistent with enhanced resistance of *gte4* mutant, previous studies demonstrate that *gte4* mutant is deficient in development due to delayed mitotic cell cycle procedure; however, the mechanism is unrevealed [36, 37]. Our data suggest that GTE4 may maintain proper cell cycle through two approaches. First, GTE4 ensures high expression of genes involved in ribosome biogenesis, which is required for active cell division. Second, it is reported that JA treatment is capable of slowing down cell cycle by arresting cells in G1 phase prior to the S-phase transition [46]. Indeed, RT-qPCR showed that cell cycle related genes are downregulated in *gte4* mutant (Additional file 10: Fig S5). Thus, the function of GTE4 in inhibiting JA signaling pathway may protect proper cell cycle procedure from being arrested.

### Noncanonical functions of GTE4-regulated JA biosynthesis in plant defense and development

JA and SA are both important defense-related plant hormone but normally act against different kinds of stresses. JA is mainly involved in response to insects, wounding and necrotrophic fungal pathogen, while SA mainly participates in response to biotrophic or hemi-biotrophic bacterial pathogen [11]. Enhanced JA usually causes enhanced resistance to fungi and more sensitive to bacteria. In this study, we found noncanonical functions of JA signaling in *gte4*. Increased JA content and JA-responsive gene expression is coupled with increased resistance to hemi-biotrophic bacterial pathogen *Pst* DC3000 and overexpression of *PR1*, which indicates that SA signaling might be activated in *gte4*. The noncanonical role of *gte4*-resulted JA signaling is also evidenced by early flowering phenotype of *gte4* mutant, because canonical JA signaling represses floral transition. Interestingly, exogenous application of SA also promotes flowering [54]. Taken together, over-accumulated JA content in *gte4* might activated SA signaling in a noncanonical pathway. However, the mechanism requires further investigation.

### Multifaced functions of GTE4 in regulating gene expression

We have profiled the genome-wide occupancy of GTE4 and found that the majority of GTE4-bound genes are downregulated in *gte4* mutant, indicating that GTE4 may mainly function as activator of gene expression. Consistently, GTE4 enrichment is positively correlated with gene expression level and active histone marks. However, we also find that small portion of GTE4-bound genes upregulated in *gte4*, suggesting that GTE4 also represses gene expression in particular circumstances. The multifaced functions of GTE4 in regulating gene expression might be achieved by interacting with different transcriptional regulators (Fig. 8). One potential candidate is TRB1. Previous study and this study identified similar DNA motif in TRB1- and GTE4-enriched peaks. GTE4 is also enriched in TRB1-peaks (Fig. 3E), suggesting that GTE4 and TRB1 may co-regulate the same targets. Interestingly, TRB1 could regulate gene expression by recruiting PRC2 and PEAT complex [44, 55], indicating potential regulatory mechanisms of GTE4. Besides CRF10 and ARID3, we identified multiple conserved DNA motifs bound by different kinds of transcription factors in GTE4-enrihced peaks (Additional file 11: Fig. S6), raising the possibility that GTE4 may carry out different functions by interacting with respective transcription factors. Given that GTE4 is a potential histone acetylation binding protein, it is not surprising that GTE4 facilitates gene expression. However, it will be interesting to find out the mechanisms that GTE4 represses gene expression.

## Conclusions

Herein, we demonstrated the functions of bromodomain-containing protein GTE4 in regulating noncanonical JA-mediated immune response. Biochemical and genetic evidences show that GTE4 attenuates immune response through repressing expression of JA biosynthesis gene *OPR3*. Elevated JA biosynthesis in *gte4* unexpectedly activated SA-mediated resistance to *Pst* DC3000. GTE4 also maintains high expression levels of ribosome biogenesis related genes during pathogen infection, which may further attenuate plant defense system. In summary, our work provides a new example of noncanonical JA-mediated pathogen resistance that involves chromatic regulator. Future studies will be required to dissect the detailed mechanisms of JA signaling in *gte4.*

## Methods

### Plant materials and growth conditions

*Arabidopsis thaliana* ecotype Columbia-0 (Col-0) was used as wild type (WT) for all experiments. The T-DNA insertion line of *gte4* (SALK_083697) was obtained from Arabidopsis Biological Resource Center (https://abrc.osu.edu/). All plants were grown at 22 °C under long-day condition (16 h light/8 h dark) and 50% humidity conditions unless otherwise specified.

### Plasmid construction and generation of transgenic plants

Genomic DNA of GTE4 with its 1 kb promoter was cloned into PC1300-TB-bHA vector modified from pCAMBIA1300 to create epitope-tagged HA fusions and then transformed by *Agrobacterium*-dipping into *gte4* mutants. Detailed information for primers can be found in Additional file 12: Table S6.

### *Pseudomonas syringae pv. tomato (Pst)* DC3000 treatment and growth assay

*Pst* DC3000 was grown on King’s B medium plate for 2 days at 28 °C. Single clone was inoculated in King’s B broth and cultured overnight at 28 °C. In the next morning, bacteria were suspended in 10 mM MgCl_2_ to OD_600_ of 0.2 then diluted 100 times. Four-week-old plants grown under long-day photoperiods and 50% humidity conditions were injected the bacterial suspension with needless syringe. Afterwards, plants were covered to keep humidity and incubated in growth chamber. Bacteria growth was analyzed at 3 days post inoculation (dpi). Proper amount of plants was pooled together as one biological replicate and six such biological replicates were measured at the same time.

### RNA extraction, quantitative RT-PCR, and high-throughput sequencing

Four-week-old plants were used for total RNA extraction by Trizol Reagent (Invitrogen, 15596026). RNA was treated with DNase before used for cDNA synthesis by QPCR cDNA synthesis kit (Vazyme, R312-02). RT-qPCR was performed on CFX96™Real-Time System (BioRad) using 2 × Universal SYBR Green Fast QPCR Mix (ABclonal, RK21203). *MON1* (*MONENSIN SENSITIVITY1*, AT2G28390), instead of *ACTIN*, was used as internal control [56, 57].

For RNA sequencing, total RNA was extracted from 4-week-old plants using Trizol Reagent and then purified by Quick-RNATM Plant MiniPrep Kit (ZYMO RESEARCH, R2024). cDNA libraries were constructed using Library Preparation VAHTS mRNA Capture Beads (Vazyme, N401-01) and VAHTS Universal V8 RNA-seq Library Prep Kit for Illumina (Vazyme, NR605-01). All samples were run on Illumina NovaSeq 6000 platform. Three biological replicates were performed for both WT and *gte4* mutant.

### Chromatin immunoprecipitation (ChIP)

ChIP of GTE4-HA was performed as previously described [58] with modifications. A 10-g mixture of leaves were divided into five parts and ground into powder in liquid nitrogen and cross-linked in Nuclear Isolation Buffer (10 mM HEPES pH 8, 1 M Sucrose, 5 mM KCl, 5 mM MgCl_2_, 0.6% Triton X-100, 0.4 mM PMSF, and 1 × Mini-Complete cocktail) with 1% formaldehyde for 15 min at room temperature with rotation. Chromatin pellet was washed with 1 mL ChIP Buffer 2 (10 mM HEPES pH 8, 0.25 M sucrose, 10 mM MgCl_2_, 1% Triton X-100, 1 mM EDTA, 5 mM β-mercaptoethanol, 1 × Mini-Complete cocktail) then resuspended with 300 μL nuclear lysis buffer (50 mM Tris–HCl pH 8, 10 mM EDTA, 1% SDS, 0.1 mM PMSF, 1 × Mini-Complete cocktail) and kept on ice for 10 min. The lysates were diluted tenfold with ChIP dilution buffer (1.1% Triton X-100, 1.2 mM EDTA, 16.7 mM Tris–HCl pH 8, 167 mM NaCl, 0.1 mM PMSF, and 1 × Mini-Complete cocktail) and sheared by sonication. After centrifugation at 12,000 rpm for 10 min, the supernatant was incubated with 3 μL anti-HA antibody (Cell Signaling Technology, 3724, C29F4) overnight with rotation at 4 °C. Then, add 40 μL Protein A/G beads (SMART Lifesciences, SA032005) into supernatant and incubated 4 h with rotation at 4 °C. High salt wash was replaced by another low salt buffer wash. DNA–protein complex was released and reverse cross-linked by boiling at 95 °C for 10 min in 100 μL 10% Chelex (BioRad, 1,422,822) with 1000 rpm shaking. After proteinase K and RNase treatment, DNA was purified by standard phenol–chloroform method. Ten and 2 g of leaves were used for sequencing and qPCR assay, respectively.

### Extraction and measurement of JA

One-gram powder of well-ground leaves was transferred into 15 mL centrifuge tube, and 20 ng dihydro-JA (H_2_JA) was added as an internal standard. The samples were vortexed for 1 min and then extracted with 5 mL methanol at 4 °C for 24 h. After centrifugation at 13,000 rpm at 4 °C for 10 min, the supernatant was transferred to a new tube, and dried with nitrogen, and then re-dissolved with 10 mL dichloromethane/methanol (v/v = 99:1) by vortexing for 1 min. JA was further purified with SPE-NH2 Cartridges (Amicrom, QNH2005). Column was activated with 6 mL dichloromethane/methanol (v/v = 99:1) before use. JA was eluted twice with 2% acetic acid/methanol. After being dried with nitrogen, JA was dissolved with 1 mL 0.05% acetic acid aqueous solution/acetonitrile (v/v = 80:20) by vortexing for 2 min and then filtered with 0.22 μm syringe filter.

Ten microliter sample was fractionated with an Acclaim C18 column (Thermo Scientific, 059142) using a Dionex UltiMate 3000 HPLC system (Thermo Scientific) at a flow rate of 0.5 mL/min. A-eluent was Milli-Q water containing 1 ‰ (v/v) formic acid, and the B-eluent was acetonitrile containing 1 ‰ (v/v) formic acid. The program of elution started at 95% (v/v) Solvent A and ramped from 5 to 20% (v/v) Solvent B over 7 min, 20 to 50% (v/v) Solvent B over 5 min, 50 to 100% (v/v) Solvent B over 5 min, remained isocratic at 100% (v/v) Solvent B over 4 min, ramping from 100 to 5% (v/v) Solvent B over 0.1 min, and remaining isocratic at 95% (v/v) Solvent A over 3.9 min. The total run time was 25 min and data were collected for the first 17 min. The chromatograms were acquired at 280 nm, and photodiode array spectra were recorded from 180 to 400 nm. Heated ESI source and interface conditions were operated in positive ion mode as follows: vaporizer temperature 400 °C, source voltage 3 kV, sheath gas 60 au, auxiliary gas 20 au, capillary temperature 380 °C, capillary voltage 6 V, and tube lens 45 V. An LTQ-Orbitrap-XL (Thermo Fisher) mass spectrometer was operated using LTQ Tune Plus v. 2.5.5 SP1 and Xcalibur software (version 2.1.0.1140), with additional analyses using the QualBrowser feature of Xcalibur.

JA standard was serially diluted to 2, 5, 10, 20, 50, and 100 ng/mL and mixed with 20 ng/mL H_2_JA as internal standard. The measured JA/H_2_JA ratio and theoretic JA/H_2_JA ratio of each standard sample was used to draw standard curve. Three biological replicates were performed for both WT, *gte4* mutant and GTE4-HA transgenic plants.

### Data analysis

Raw data were filtered using Cutadapt (v2.7) and were checked for quality scores and other metrics using FastQC (v0.11.9). ChIP-seq data were aligned by Bowtie2 (v2.3.5) with the default parameters. The mapped reads were sorted, and duplicates were removed using SAMtools (v1.9). MACS2 (v2.2.7) was used for Peak calling with *P* = 1.0e − 5. DNA motif identification was performed with Homer (v4.11). In GTE4 binding profiling, the number of mapped ChIP-seq reads in 50-bp bins normalized to the total number of reads. The metaplots were built by Deeptools (v3.5.0). The R package clusterProfiler (v4.0.5) was used to perform the GO enrichment analysis involved in the biological processes. Other analysis and figures were done using R (v4.0.3). Accession numbers of published ChIP-seq data of histone modifications used in this study are listed in Additional file 3: Table S2. For differential expression analysis in RNA-seq data, HISAT2 (v2.1.0) was used for sequence alignment; StringTie (v2.0.6) was used for quantification of expression level. Genes showing a *P* < 0.05 were considered as significantly differential expressed genes.

## Supplementary Information

**Additional file 1: Fig. S1.** Characterizations of *gte4* mutant and GTE4-HA rescue plants. (A)Diagram of *gte4 *T-DNA insertionmutant. (B) Genotyping of* gte4* T-DNAinsertion mutant. (C) RT-PCR verification of *GTE4* transcripts in *gte4*T-DNA insertion mutant. *MON1* servesas internal control. (D) Diagram of GTE4-HA construct for rescuing *gte4* mutant. (E) Immunoblots of GTE4-HAprotein in GTE-HA rescue plants.

**Additional file 2: Table S1.** List of peaks and geneswith GTE4 enrichment.

**Additional file 3: Table S2.** Published ChIP-seq dataused in this study.

**Additional file 4: Fig. S2.** Representative snapshots ofIGV views of GTE4 enrichment on ribosome biogenesis related genes.

**Additional file 5: Table S3.** List of differentiallyexpressed genes in *gte4* under controlcondition.

**Additional file 6: Table S4.** List of differentiallyexpressed genes in *gte4* under *Pst* DC3000 condition.

**Additional file 7: Fig. S3.** RT-qPCR verifying theexpression of GTE4-bound and downregulated genes in *gte4* under normal condition. Data are presented as mean±SEM of 2biological replicates.

**Additional file 8: Fig. S4.** JA-responsive genes areover-activated while growth pathways are impaired in *gte4*. (A) Biological repeat of RT-qPCR verification ofJA-responsive gene expression in *gte4*.(B, C) GO analysis of downregulated genes in *gte4* under control (B) and *Pst*DC3000 treatment (C) conditions.

**Additional file 9: Table S5.** List of genes with GTE4enrichment and co-upregulated in *gte4*under both conditions.

**Additional file 10: Fig. S5.** RT-qPCR verification ofcell cycle related gene expression in *gte4*.Data are presented as mean±SEM of 2 biological replicates.

**Additional file 11: Fig. S6.** Examples of conservedtranscription factor binding motifs identified in GTE4-enriched peaks.

**Additional file 12: Table S6.** Primers used in thisstudy.

**Additional file 13: Fig. S7.** Uncropped pictures for Additional file 1_Fig S1. A Picture for Fig S1B. B Pictures for Fig S1C. Dash-lineboxes indicate samples presented in Fig S1C. C Pictures for Fig S1E.

## Data Availability

The datasets presented in this study can be found in online repositories. The names of the repository/repositories and accession number(s) can be found below: https://www.ncbi.nlm.nih.gov/bioproject/?term=PRJNA776416. All data generated or analyzed during this study are included in this published article, its supplementary information files, and publicly available repositories.

## Abbreviations

JA: Jasmonic acid
SA: Salicylic acid
GTE4: Global transcription factor group E 4
ChIP: Chromatin immunoprecipitation
SEM: Standard error of mean
OPR3: Oxophytodienoate-Reductase 3
