# Supplementary figures and images for: Bromodomain-containing factor GTE4 regulates Arabidopsis immune response
Source: BMC Biol. 2022 Nov 13;20:256. doi: 10.1186/s12915-022-01454-5 (PMC9655792; doi:10.1186/s12915-022-01454-5)

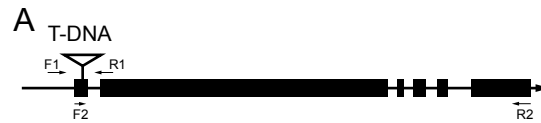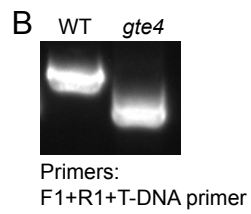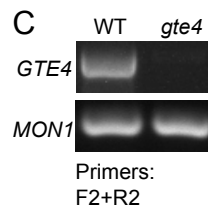

**D**

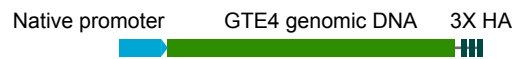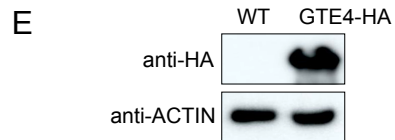

Supplement: Supplementary file 1 — Additional file 1: Fig. S1. Characterizations of gte4 mutant and GTE4-HA rescue plants. (A)Diagram of gte4 T-DNA insertionmutant. (B) Genotyping of gte4 T-DNAinsertion mutant. (C) RT-PCR verification of GTE4 transcripts in gte4T-DNA insertion mutant. MON1 servesas internal control. (D) Diagram of GTE4-HA construct for rescuing gte4 mutant. (E) Immunoblots of GTE4-HAprotein in GTE-HA rescue plants. [file 12915_2022_1454_MOESM1_ESM.pdf]

### rRNA processing related

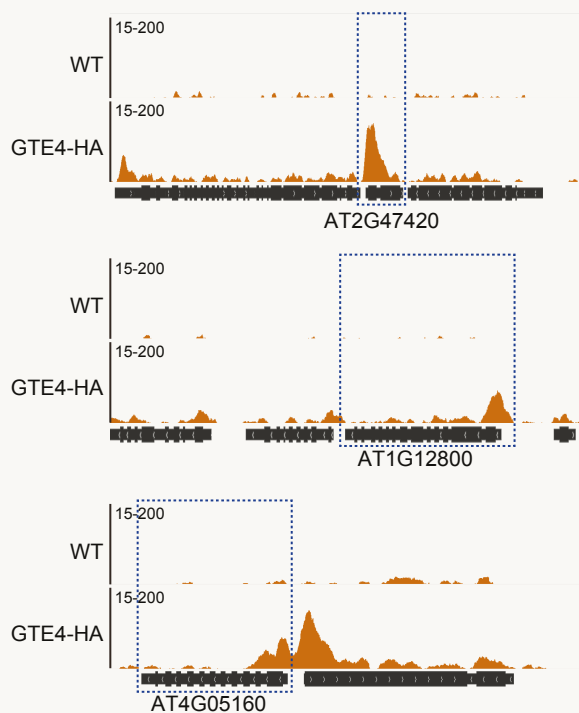

### Ribosomal protein related

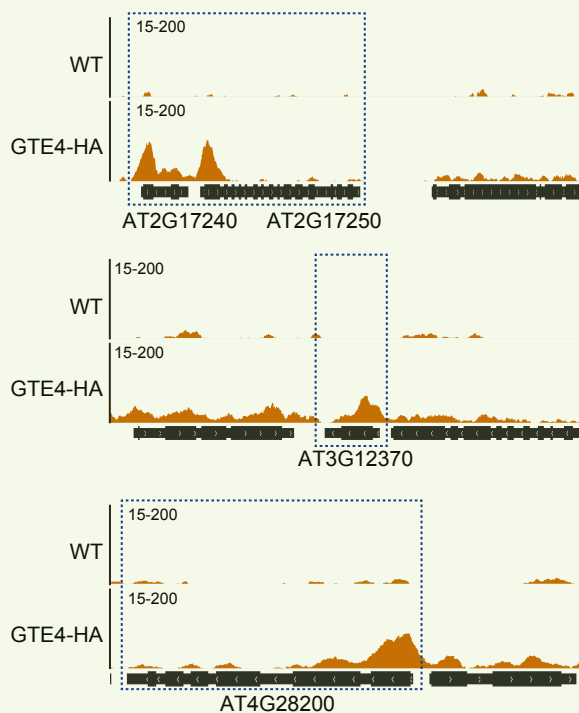

Supplement: Supplementary file 4 — Additional file 4: Fig. S2. Representative snapshots ofIGV views of GTE4 enrichment on ribosome biogenesis related genes. [file 12915_2022_1454_MOESM4_ESM.pdf]

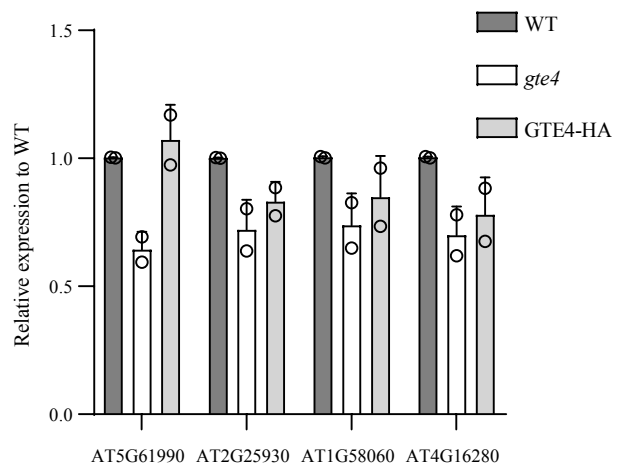

Supplement: Supplementary file 7 — Additional file 7: Fig. S3. RT-qPCR verifying theexpression of GTE4-bound and downregulated genes in gte4 under normal condition. Data are presented as mean±SEM of 2biological replicates. [file 12915_2022_1454_MOESM7_ESM.pdf]

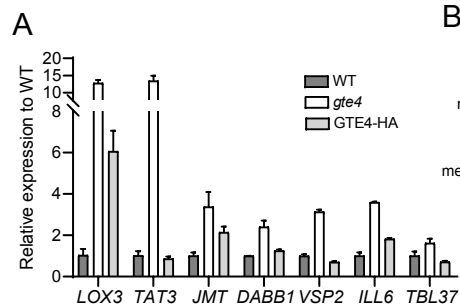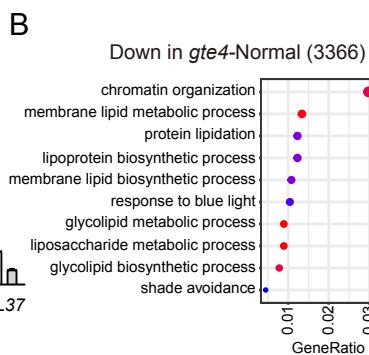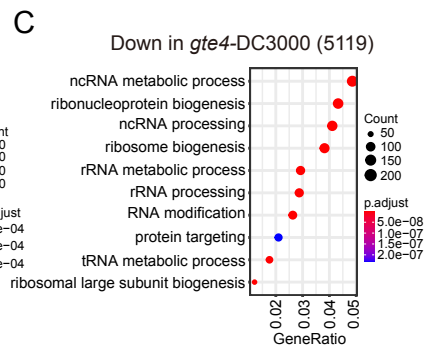

Supplement: Supplementary file 8 — Additional file 8: Fig. S4. JA-responsive genes areover-activated while growth pathways are impaired in gte4. (A) Biological repeat of RT-qPCR verification ofJA-responsive gene expression in gte4.(B, C) GO analysis of downregulated genes in gte4 under control (B) and PstDC3000 treatment (C) conditions. [file 12915_2022_1454_MOESM8_ESM.pdf]

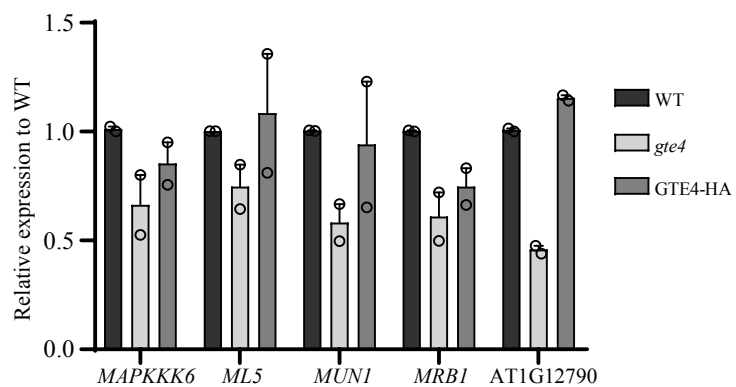

Supplement: Supplementary file 10 — Additional file 10: Fig. S5. RT-qPCR verification ofcell cycle related gene expression in gte4.Data are presented as mean±SEM of 2 biological replicates. [file 12915_2022_1454_MOESM10_ESM.pdf]

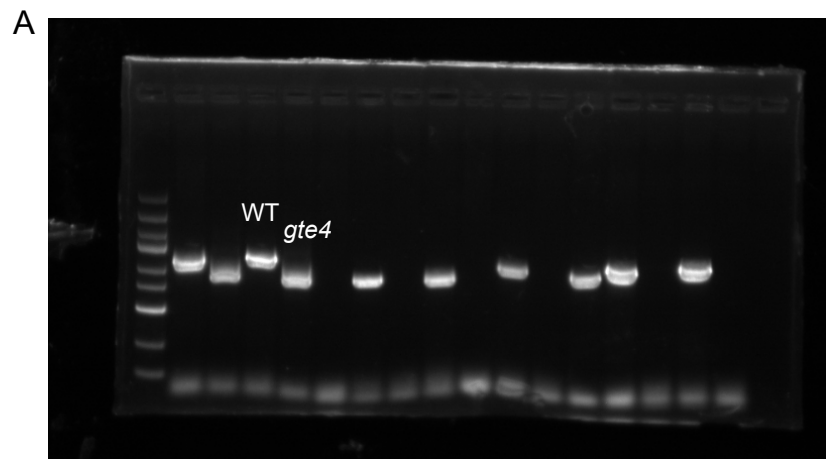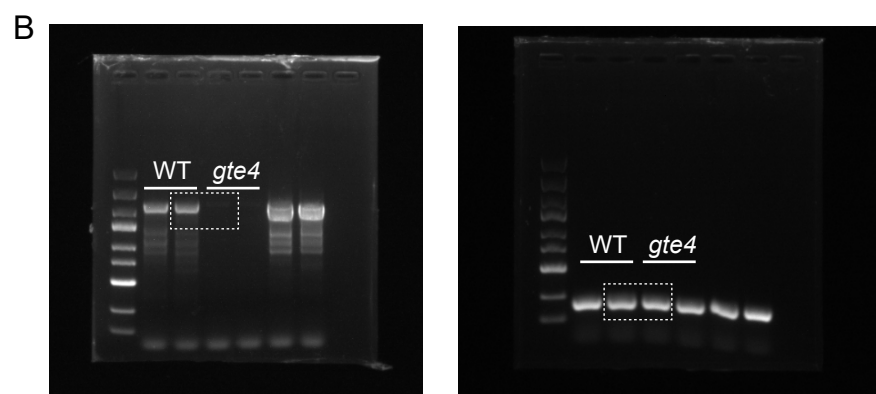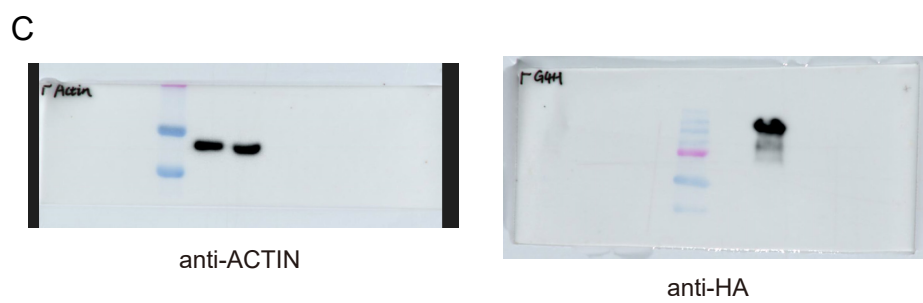

Supplement: Supplementary file 13 — Additional file 13: Fig. S7. Uncropped pictures for Additional file 1_Fig S1. A Picture for Fig S1B. B Pictures for Fig S1C. Dash-lineboxes indicate samples presented in Fig S1C. C Pictures for Fig S1E. [file 12915_2022_1454_MOESM13_ESM.pdf]
