# Supplementary material for: Bromodomain-containing factor GTE4 regulates Arabidopsis immune response
Source: BMC Biol. 2022 Nov 13;20:256. doi: 10.1186/s12915-022-01454-5 (PMC9655792; doi:10.1186/s12915-022-01454-5)
Supplement: Supplementary file 3 — Additional file 3: Table S2. Published ChIP-seq dataused in this study. [file 12915_2022_1454_MOESM3_ESM.docx]

Table S2. Public ChIP-seq data used in this study

| **ChIP-seq** | **Accession Number** |
| --- | --- |
| H3K27ac | GSE80056 |
| H3K4me3 | GSE22276 |
| H3K9ac | GSE22276 |
| H4K5ac | GSE161515 |
| H4K8ac | GSE161515 |
