# Supplementary material for: Bromodomain-containing factor GTE4 regulates Arabidopsis immune response
Source: BMC Biol. 2022 Nov 13;20:256. doi: 10.1186/s12915-022-01454-5 (PMC9655792; doi:10.1186/s12915-022-01454-5)
Supplement: Supplementary file 11 — Additional file 11: Fig. S6. Examples of conservedtranscription factor binding motifs identified in GTE4-enriched peaks. [file 12915_2022_1454_MOESM11_ESM.pdf]

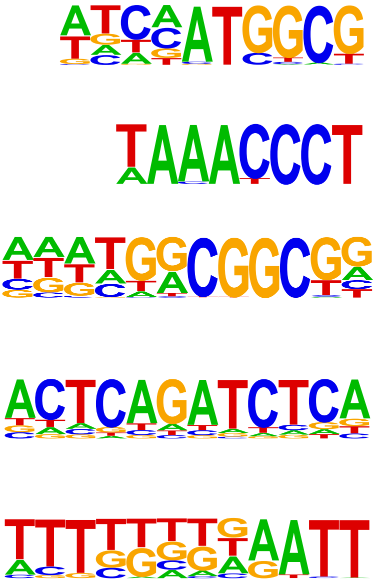

YY2 (*P*=1e-54)

TRP2 (*P*=1e-51)

ERF4 (*P*=1e-45)

GATA11 (*P*=1e-35)

HOXD3 (*P*=1e-22)
