# Supplementary material for: Bromodomain-containing factor GTE4 regulates Arabidopsis immune response
Source: BMC Biol. 2022 Nov 13;20:256. doi: 10.1186/s12915-022-01454-5 (PMC9655792; doi:10.1186/s12915-022-01454-5)
Supplement: Supplementary file 12 — Additional file 12: Table S6. Primers used in thisstudy. [file 12915_2022_1454_MOESM12_ESM.docx]

Table S6. Primers used in this study

| No. | Name | Sequence |
| --- | --- | --- |
| PR162 | MON1-RT-qPCR-F | GGATTGGGACCCCACAAGAC |
| PR163 | MON1-RT-qPCR-R | TCACCAAGAGAAAGGACTAGCTCC |
| PR174 | TA3-ChIP-qPCR-F | TGGAATCTCAGGGTCAAGG |
| PR175 | TA3-ChIP-qPCR -R | CCTTCTGAGGTGAGGGACA |
| PR182 | GTE4-F | CAGCTATGACCATGATTACGAGCTATGCATGTCATTCAATATAGAC |
| PR183 | GTE4-R | ATCCCCGGGTACCGAGCTCGAATTCGGTCTGATCTGATCCAGATGATG |
| PR416 | *gte4*-SALK_083697-F | AGAGCTTCTTTATGATATACGCGAA |
| PR417 | *gte4*-SALK_083697-R | CATCAGTGGTATCTCTTTCCACAAA |
| PR1997 | OPR3-P2-ChIP-qPCR-F | CCCACATGTGCCTGGAATCT |
| PR1998 | OPR3-P2-ChIP-qPCR-R | AGCACGTCCAACATGCCATA |
| PR2039 | LOX3-RT-qPCR-F | CACACTTAAGCCGGTAGCCA |
| PR2040 | LOX3-RT-qPCR-R | ACTGGAGGTGTAAGCACACG |
| PR2041 | TAT3-RT-qPCR-F | TGCTCGTGCTGTCTATAGCG |
| PR2042 | TAT3-RT-qPCR-R | CGACGGTATTCTCATCCGCA |
| PR2043 | JMT-RT-qPCR-F | CTTGTGTCCTGACCTCGACC |
| PR2044 | JMT-RT-qPCR-R | ACCCGGTCGTAAAACTCTGG |
| PR2045 | DABB1-RT-qPCR-F | TCTCGACCAAGTGCTTCACC |
| PR2046 | DABB1-RT-qPCR-R | GGACGTGAGTGAAGTCGGAG |
| PR2079 | AT1G48830-RT-qPCR-F | TGCTCAGGCTTTCTTTGATTTGG |
| PR2080 | AT1G48830-RT-qPCR-R | ACAACAATTGCCTTGCGTCC |
| PR2085 | SVR1-RT-qPCR-F | CGACATCTCCTTCGCTCCTC |
| PR2086 | SVR1-RT-qPCR-R | TGGAGTTTGAGAGTGCCGTC |
| PR2087 | AT2G44120-RT-qPCR-F | AGTGGTTCCAGAGTCTGTGC |
| PR2088 | AT2G44120-RT-qPCR-R | AAGATAAGCTTGCGGGCCTC |
| PR2089 | AT2G47610-RT-qPCR-F | GCCCCGAAGAAAGGAGTGAA |
| PR2090 | AT2G47610-RT-qPCR-R | ACCGAATTGCTTAGGCCTCC |
| PR2091 | AT3G01160-RT-qPCR-F | GATCCAGTGGAGTCGCAGAG |
| PR2092 | AT3G01160-RT-qPCR-R | GAATTTAGGGTCCGTGTGCG |
| PR2093 | RNC4-RT-qPCR-F | TTTATCGGCGAGCTCCACTG |
| PR2094 | RNC4-RT-qPCR-R | AACTTTTCGCGGCGCATTAG |
| PR2099 | CMAL-RT-qPCR-F | TGCGGAAGCAGTGGTCAATA |
| PR2100 | CMAL-RT-qPCR-R | TCACAGGCACATGTGAGGAC |
| PR2101 | AT5G20290-RT-qPCR-F | CAGCCAACACCAAGCTCTCA |
| PR2102 | AT5G20290-RT-qPCR-R | AGTATCGAGCCTCAACGCAC |
| PR2106 | AtNUG2-RT-qPCR-F | CGATGCGAATCGTGCTGATG |
| PR2107 | AtNUG2-RT-qPCR-R | AAGGATTTTACCACCGGCGT |
| PR2111 | VSP2-RT-qPCR-F | TGACCGTTGGAAGTTGTGGA |
| PR2112 | VSP2-RT-qPCR-R | CGAACCATTAGGCTTCAATATGAGA |
| PR2113 | ILL6-RT-qPCR-F | ATGAGTGTCGGGTTTGGACC |
| PR2114 | ILL6-RT-qPCR-R | CTCCGGGTTCTCATGGATCG |
| PR2115 | TBL37-RT-qPCR-F | GCACCGTCCCAAGGTTTGAT |
| PR2116  PR2176  PR2177 | TBL37-RT-qPCR-R  OPR3-P1-ChIP-qPCR-F  OPR3-P1-ChIP-qPCR-R | CAGTGAGTCACCCACGAACA  CGCGTTTGCGTTTTCAGAGT  GGAGTGGTCCGTTGAGCATAA |
